# Infant Formula With a Specific Blend of Five Human Milk Oligosaccharides Drives the Gut Microbiota Development and Improves Gut Maturation Markers: A Randomized Controlled Trial

**DOI:** 10.3389/fnut.2022.920362

**Published:** 2022-07-06

**Authors:** Miroslava Bosheva, Istvan Tokodi, Aleksander Krasnow, Helle Krogh Pedersen, Oksana Lukjancenko, Aron C. Eklund, Dominik Grathwohl, Norbert Sprenger, Bernard Berger, Colin I. Cercamondi, 5 HMO Study Investigator Consortium, Viktor Bauer

**Affiliations:** Dr. Kenessey Albert Hospital and Clinic, Balassagyarmat, Hungary; Polyclinic of Gynecology and Obstetrics Arciszewscy, Bialystok, Poland; University Multiprofile Hospital for Active Treatment Deva Mariya—Neonatology, Burgas, Bulgaria; Medical Center Prolet—Pediatrics department, Ruse, Bulgaria; Medical Center Excelsior, Sofia, Bulgaria; Multiprofile Hospital for Active Treatment Sveti Ivan Rilski, Kozloduy, Bulgaria; Medical Center PROMED, Krakow, Poland; Medical Center Pratia Warszawa, Warszawa, Poland; College of Medical Sciences, University of Rzeszów, Rzeszów, Poland; Medical Center-1, Sevlievo, Bulgaria; Individual Practice for Specialized Medical Assistance, Stara Zagora, Bulgaria; Primary Health Care Clinic Clinical Vitae, Gdansk, Poland; ALERGO-MED Specialist Medical Clinic, Tarnow, Poland; Futurenest Clinical Research, Miskolc, Hungary; Medical Center Clinexpert, Budapest, Hungary; Dr. Jan Biziel’s University Hospital No. 2, Bydgoszcz, Poland; Plejady Medical Center, Krakow, Poland; Medical Center Sveti Ivan Rilski Chudotvorets, Blagoevgrad, Bulgaria; Center of Innovative Therapies, Piaseczno, Poland; Medical Center Pratia Ostroleka, Ostroleka, Poland; Kanizsai Dorottya Hospital, Nagykanizsa, Hungary; Diagnostic Consultative Center Ritam, Stara Zagora, Bulgaria; Multiprofile Hospital for Active Treatment Sveti Georgi, Montana, Bulgaria; Alitera Medical Centre, Sofia, Bulgaria; Family Pediatric Surgery/Babadoki Ltd., Szeged, Hungary; Policlinic Bulgaria—Department of pediatrics; Sofia, Bulgaria; Non-public Health Care Institution Specialist Clinics ATOPIA, Krakow, Poland; Bugát Pál Hospital—Department of Pediatrics, Gyöngyös, Hungary; Medical Center—Izgrev Ltd., Sofia, Bulgaria.; ^1^University Multiprofile Hospital for Active Treatment, St. George Medical University, Plovdiv, Bulgaria; ^2^Infant and Children’s Department, St. George’s Hospital, Székesfehérvár, Hungary; ^3^Gdansk Health Center, Gdańsk, Poland; ^4^Clinical Microbiomics, Copenhagen, Denmark; ^5^Biostatistics and Data, Nestlé Research, Lausanne, Switzerland; ^6^Nestlé Institute of Health Sciences, Nestlé Research, Lausanne, Switzerland; ^7^Nestlé Product Technology Center – Nutrition, Société des Produits Nestlé S.A., Vevey, Switzerland

**Keywords:** human milk oligosaccharides (HMOs), infant formula, gut microbiota, bifidobacteria, *Bifidobacterium longum* subsp. *infantis* (*B. infantis*), *Clostridioides (C.) difficile*, intestinal immune response, gut maturation

## Abstract

**Background:**

Human milk oligosaccharides (HMOs) have important biological functions for a healthy development in early life.

**Objective:**

This study aimed to investigate gut maturation effects of an infant formula containing five HMOs (2′-fucosyllactose, 2′,3-di-fucosyllactose, lacto-N-tetraose, 3′-sialyllactose, and 6′-sialyllactose).

**Methods:**

In a multicenter study, healthy infants (7–21 days old) were randomly assigned to a standard cow’s milk-based infant formula (control group, CG); the same formula with 1.5 g/L HMOs (test group 1, TG1); or with 2.5 g/L HMOs (test group 2, TG2). A human milk-fed group (HMG) was enrolled as a reference. Fecal samples collected at baseline (*n*∼150/formula group; HMG *n* = 60), age 3 (*n*∼140/formula group; HMG *n* = 65) and 6 (*n*∼115/formula group; HMG *n* = 60) months were analyzed for microbiome (shotgun metagenomics), metabolism, and biomarkers.

**Results:**

At both post-baseline visits, weighted UniFrac analysis indicated different microbiota compositions in the two test groups (TGs) compared to CG (*P* < 0.01) with coordinates closer to that of HMG. The relative abundance of *Bifidobacterium longum* subsp. *infantis* (*B. infantis*) was higher in TGs vs. CG (*P* < 0.05; except at 6 months: TG2 vs. CG *P* = 0.083). *Bifidobacterium* abundance was higher by ∼45% in TGs vs. CG at 6-month approaching HMG. At both post-baseline visits, toxigenic *Clostridioides difficile* abundance was 75–85% lower in TGs vs. CG (*P* < 0.05) and comparable with HMG. Fecal pH was significantly lower in TGs vs. CG, and the overall organic acid profile was different in TGs vs. CG, approaching HMG. At 3 months, TGs (vs. CG) had higher secretory immunoglobulin A (sIgA) and lower alpha-1-antitrypsin (*P* < 0.05). At 6 months, sIgA in TG2 vs. CG remained higher (*P* < 0.05), and calprotectin was lower in TG1 (*P* < 0.05) vs. CG.

**Conclusion:**

Infant formula with a specific blend of five HMOs supports the development of the intestinal immune system and gut barrier function and shifts the gut microbiome closer to that of breastfed infants with higher bifidobacteria, particularly *B. infantis*, and lower toxigenic *Clostridioides difficile*.

**Clinical Trial Registration:**

[https://clinicaltrials.gov/ct2/show/], identifier [NCT03722550].

## Introduction

In human milk, the third largest solid component after lactose and lipids is a group of over 160 structurally diverse oligosaccharides known as human milk oligosaccharides (HMOs) (1, 2). Three main categories of HMOs are generally described including neutral fucosylated [e.g., 2′fucosyllactose (2′FL) and 2′,3-di-O-fucosyllactose (DFL)], neutral non-fucosylated [e.g., lacto-N-tetraose (LNT)], and acidic sialylated [e.g., 3′sialyllactose (3′SL) and 6′sialyllactose (6′SL)] oligosaccharides (3–6). HMOs play diverse and important roles in the development of infants starting with their prebiotic function, which supports the establishment and maintenance of a balanced gut microbiota (2, 6–8). HMOs have been long recognized to drive the *Bifidobacterium* dominance in breastfed infants. Not all members of the *Bifidobacterium* genus can metabolize HMOs. *Bifidobacterium longum* subsp. *infantis* (*B. infantis*) is known as a dedicated HMO consumer and able to proliferate in the presence of HMOs (7). Additionally, a role of HMOs in immune protection has been demonstrated *via* anti-adhesive antimicrobial effects (8–10), regulation of intestinal epithelial cell response (6, 7), and modulation of immune responses *via* direct effects on immune cells and cytokine secretion (11–13). Furthermore, potential benefits of HMOs on brain development have been reported (14, 15).

Breastfeeding is the reference for infant nutrition, and the lack of HMOs in infant formula is likely one of the factors contributing to differences in health outcomes that have been observed between breastfed and formula-fed infants (16). Advances in technology now allow the synthesis of HMOs, and some of them are being added to formulas to provide infants who cannot be fed with human milk with identical oligosaccharides to those found in human milk (7). Clinical trials have demonstrated safety and good tolerance of infant formulas supplemented with 2′FL alone (17, 18), the combination of 2′FL and lacto-N-neotetraose (LNnT) (19, 20), and a blend of five HMOs (21). HMOs have been shown to play an important role in the development of the intestinal microbiome in breastfed infants (22, 23), and more recently, infant formula with 2′FL and LNnT was shown to promote a microbiome more similar to the human milk-fed reference than formula not containing HMOs (24). In another trial, infants fed a formula containing 2′FL and galacto-oligosaccharides had plasma and *ex vivo* inflammatory cytokine profiles more similar to the breastfed reference than control infants fed a formula with galacto-oligosaccharides alone (25).

With more HMOs becoming available, it is possible to supplement formulas with blends of HMOs providing structurally diverse and complex oligosaccharides to formula-fed infants. Our study evaluated for the first time the gut maturation effects (microbiota, metabolites, and selected maturation markers) of an infant formula containing a specific blend of five HMOs (2′FL, DFL, LNT, 3′SL, and 6′SL) that is based on the HMO profile found in human milk and covers the major HMOs from all three categories. We hypothesized that infants receiving a starter formula with the five-HMO blend for 6 months would have a gut microbiota composition and metabolic activity more similar to that observed in human milk-fed infants and improved gut maturation markers than their control peers. This article reports the secondary outcomes up to 6 months of age of a 15-month study.

## Subject and Methods

### Study Design

This randomized, controlled, double-blind trial conducted between September 2018 and November 2021 at 32 study sites in Bulgaria, Hungary, and Poland consisted of three randomized formula-fed groups and a non-randomized human milk-fed group (HMG) as reference. The study was conducted according to the Declaration of Helsinki and the International Conference on Harmonization Guidelines for good clinical practice. All procedures involving human subjects were approved by the Scientific and Research Ethics Committee of Medical Research Council (Budapest, Hungary), the Bioethics Committee at the Regional Medical Chamber (Gdańsk, Poland), and the Ethics Committee of the Scientific Research at Medical University (Sofia, Bulgaria). Written informed consent was obtained prior enrollment from the parents of all infants. The trial was registered on ClinicalTrials.gov as NCT03722550.

The overall study included a follow-up up to 15 months of age with formula-fed infants consuming a starter infant formula (IF) from enrollment to 6 months, transitioning to a follow-up formula from 6 to 12 months, and followed by growing-up milk from 12 to 15 months. This report encompasses the first 6 months of the study during which participants were expected to consume the assigned IF (or breastmilk in HMG) continuously until 6 months of age, with the addition of complementary foods permitted after 4 months of age. Subject demographic data were collected at the enrollment visit. Fecal samples were collected at enrollment (baseline), 3, and 6 months of age in a random subset of infants whose parents agreed for the stool sampling (“first-in, first-served principle”). Parents were instructed to immediately freeze the collected fecal samples in their freezer at home (∼−20°C) and to bring them within 3 days to the study site where samples were frozen at −80°C until analysis. Transport to the study sites was done in cooling bags containing sufficient cool packs to keep samples frozen.

### Study Participants

All infants were required to be healthy and full-term, with birth weight between 2,500 and 4,500 g, and aged ≥ 7– ≤21 days at enrollment. For formula-fed infants, parents had elected to formula feeding prior to enrollment. For the HMG, infants had to consume human milk exclusively from birth to enrollment, and their parents had elected to continue exclusive breastfeeding at least until 4 months of age. Infants with conditions requiring feedings other than those specified during the trial period or requiring complementary foods at or prior to enrollment were not eligible, as were infants with evidence of major congenital malformations, documented or suspected congenital infections, a history of admission to the neonatal intensive care unit for any reason except jaundice phototherapy, and participating in other clinical trials. In addition, infants having used any medication known or suspected to affect fat digestion, absorption or metabolism, stool characteristics, growth, or gastric acid secretion were not eligible.

### Interventions

The three formula-fed groups were the control group (CG) fed a standard IF without HMOs, test group 1 (TG1) fed the same standard IF with a concentration of 1.5 g/L of the five-HMO blend, and test group 2 (TG2) fed the same standard IF with a concentration of 2.5 g/L of the five-HMO blend. The concentrations of the five HMOs in TG1 and TG2 were 0.87 and 1.45 g/L for 2′FL, 0.10 and 0.14 g/L for DFL, 0.29 and 0.48 g/L for LNT, 0.11 and 0.18 g/L for 3′SL, and 0.14 and 0.24 g/L for 6′SL, respectively. These concentrations are all in the range of that reported in human milk for the individual HMOs (26–30). The standard IF was a bovine milk-based whey predominant term infant formula with 67 kcal/100 mL reconstituted formula, consisting of 1.9 g intact protein (70% whey/30% casein)/100 kcal, 11.1 g carbohydrates/100 kcal, and 5.3 g lipids/100 kcal.

Formula-fed infants were randomized to CG, TG1, or TG2 using Medidata Balance, and randomization was stratified by study center, sex, and mode of delivery with an equal study infant allocation ratio of 1:1:1 for CG:TG1:TG2. The study was double blinded with the identity of the specific product masked to all parents of enrolled infants, study investigators, and study staff using individual coding.

### Microbiome Analysis and Ecological Measures

Microbial DNA was extracted from frozen feces, purified, and shotgun sequenced with 2 × 150 bp sequencing, as described previously (15). Taxonomic relative abundances were calculated using the metagenomic species (MGS) approach, which enables the quantification of both known characterized and uncharacterized microbial species (31) (see also Supplementary Methods).

A phylogenetic tree connecting the MGSs was generated using previously identified conserved genes (Supplementary Methods). Alpha diversity as Faith’s phylogenetic diversity (PD) (32) and beta diversity as weighted UniFrac distance (33) were calculated using this tree with the *PhyloMeasures* and *phyloseq* R packages, respectively. Additional alpha diversity indexes, including richness and Shannon diversity, were calculated independently for gene, MGS, and genus. Centroids for vaginally delivered HMG infants were calculated at each timepoint using all principal coordinates analysis (PCoA) coordinates, and distance from a sample of the respective timepoint to the HMG centroid (*d*_*vaginal delivered HMG*–*centroid*_) was calculated as the Euclidean distance in this PCoA space. All distances and alpha diversity measures were calculated using rarefied abundances.

### Analysis of Fecal Biomarkers, pH, and Organic Acids

Commercially available ELISA kits were used to analyze fecal biomarkers at baseline, age 3, and 6 months, including secretory immunoglobulin A (sIgA), calprotectin (both Immundiagnostik AG, Bensheim, Germany), and alpha-1-antitrypsin (AAT; BioVendor – Laboratorni medicina a.s., Brno, Czech Republic).

Fecal pH and organic acids (including lactate, acetate, butyrate, isobutyrate, propionate, valerate, and isovalerate) were assessed at baseline, 3, and 6 months of age using pH indicator paper (pH range 1–10; Merck, Darmstadt, Germany) and validated liquid chromatography-tandem mass spectrometry according to a modified previously published method (34), respectively.

### Statistics

For weighted UniFrac analysis, permutational multivariate analysis of variance (PERMANOVA) tests assessing marginal effects of the terms were performed using the adonis2 function from the *vegan* R package with 1,000 permutations (35). Alpha diversity indexes and taxonomical abundances were compared among the groups using the Kruskal–Wallis H test with *post hoc* Dunn’s test. Fisher’s exact test was used to assess differences in taxonomical prevalence. To compare *d*_*vaginal delivered HMG*–*centroid*_ between the feeding groups, we used the Mann–Whitney U test. For the Kruskal–Wallis H test and the corresponding pairwise tests, all considered taxa detected in at least 10 formula-fed infants in the given comparison were subjected to the testing scheme. For Fisher’s exact test and the corresponding pairwise tests, we included all entities which were i) detected in ≥ 3 samples and ii) undetected in ≥ 3 samples from formula-fed infants in the given comparison. All statistical tests were run using R software (v. 4.0.3). The dataset for microbiota analyses consisted of infants who provided a stool sample at any timepoint and were compliant with the study feeding on ≥ 80% of the days. Up to 4 months of age, a compliant day was defined as a day on which the study product (or human milk in the reference group) was exclusively fed (i.e., no complementary foods or other formulas). From 4 to 6 months of age, a compliant day was defined as a day on which at least one serving of the study product was consumed. For the HMG, there were no specific requirements for the period between 4 and 6 months age. For bifidobacteria analysis, four of nine *Bifidobacterium* (sub)species were defined as infant-type *Bifidobacterium* (sub)species based on a previous publication, including *B. longum* subsp. *infantis*, *B. longum* subsp. *longum*, *B. bifidum*, and *B. breve* (36), and the sum of their relative abundance was compared between the groups.

Fecal pH, organic acids, and biomarkers were based on grams of fecal dry weight and were log-transformed for analysis if needed. Intervention differences at 3 and 6 months of age were examined in ANCOVA models adjusted for baseline values of the measure of interest or Wilcoxon test for infants providing a baseline sample and a sample at the respective timepoint. Short-chain fatty acids (SCFA; acetate, butyrate, isobutyrate, propionate, valerate, isovalerate) were analyzed as a proportion of total SCFA. Analyses were conducted using SAS/STAT software version 9.4 of the SAS System (SAS Institute Inc., Cary, NC, United States).

This article reports the secondary endpoints of a study for which the sample size calculation was based on the two co-primary endpoints: growth at 4 months and incidence of respiratory tract infections at 15 months (ClinicalTrials.gov: NCT03722550); therefore, no sample size calculation is available for the endpoints reported herein.

## Results

### Participants

**FIGURE 1:**
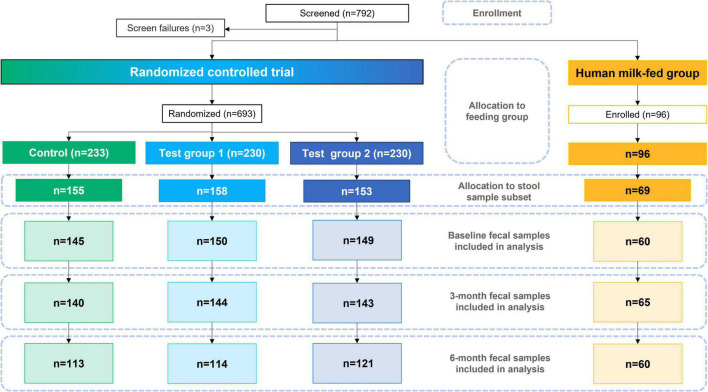
Study participant flowchart. Control was without HMOs; test group 1 was with 1.5 g HMOs/L; test group 2 with 2.5 g HMOs/L. HMOs human milk oligosaccharides.

Of the 693 randomized formula-fed infants (CG, *n* = 233; TG1, *n* = 230; TG2, *n* = 230) and 96 non-randomized human milk-fed infants in the HMG who were enrolled for the overall study, the stool samples of 535 infants were analyzed and included in this report (CG, *n* = 155; TG1, *n* = 158; TG2, *n* = 153; HMG, *n* = 69; Figure 1). Baseline characteristics of the infants included in this report are shown in Table 1 and were largely comparable among the feeding groups, except for longer maternal and paternal education and slightly older gestational age at birth in HMG compared with the formula-fed groups.

**TABLE 1:** Demographic characteristics, by feeding group (*n* = 535).

	CG (*n* = 155)	TG1 (*n* = 158)	TG2 (*n* = 153)	HMG (*n* = 69)	*P-value* ^a^
Age at baseline (days)	14.5 ± 4.6	14.7 ± 4.5	14.3 ± 4.5	15.4 ± 3.8	0.316
Sex					0.426
Male	80 (51.6%)	78 (49.4%)	77 (50.3%)	42 (60.9%)	
Female	75 (48.4%)	80 (50.6%)	76 (49.7%)	27 (39.1%)	
Gestational age at birth (weeks)	38.7 ± 1.3	38.7 ± 1.2	38.7 ± 1.1	39.1 ± 1.0	0.047
Delivery mode					0.501
Vaginal	64 (41.3%)	64 (40.5%)	63 (41.2%)	35 (50.7%)	
Cesarean	91 (58.7%)	94 (59.5%)	90 (58.8%)	34 (49.3%)	
Complementary foods by age 6 months (yes)	138 (94.5%)	144 (96.6%)^b^	144 (96.0%)	58 (87.9%)	0.079
Maternal education (years)	13.7 ± 3.7	13.8 ± 3.9	13.6 ± 3.9	16.7 ± 2.9	<0.001
Paternal education (years)	13.2 ± 3.5	12.9 ± 3.5	13.1 ± 3.9	15.6 ± 3.0	<0.001

*Data shown as mean ± SD or n (%). CG, control group; TG1, test group 1 (1.5 g HMOs/L); TG2, test group 2 (1.5 g HMOs/L); HMG, human milk-fed group; HMOs, human milk oligosaccharides.*

*^a^P-values for overall group comparison are derived from one-way ANOVA or Pearson’s chi-squared test. ^b^For 9 infants in TG1 it was not known whether complementary foods were consumed or not by age 6 months.*

### Gut Microbiome

**FIGURE 2:**
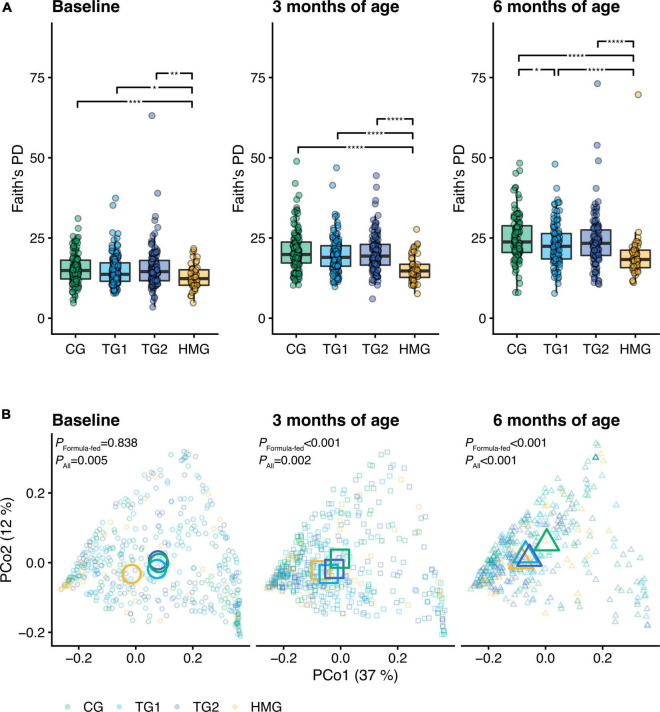
**(A)** Alpha diversity (Faith’s phylogenetic diversity) of the gut microbiota of the infants in the four feeding groups at each timepoint (baseline left, 3 months of age center, 6 months of age right). Box plots show the median and 25^th^ and 75^th^ percentiles with Tukey whiskers. Within each timepoint, all feeding groups were compared pairwise (Dunn’s test), and significant differences are highlighted with significance bars. *P < 0.05, **P < 0.01, ***P < 0.001, ****P < 0.0001. **(B)** Principal coordinates analysis (PCoA) based on weighted UniFrac distance. Feeding groups are color-coded and faceted by timepoint. Individual data points are shown, with the mean (centroid) of each group indicated as a larger symbol. The x- and y-axis labels indicate the microbial variance explained by the first two principal coordinates. P-values for permutational multivariate analysis of variance (PERMANOVA) using feeding group as explanatory variable are shown for all infants and the subset of formula-fed infants at each visit. At baseline/3/6 month of age, CG, n = 135/135/111; TG1, n = 140/138/113; TG2, n = 136/140/117; HMG, n = 50/55/50. CG, control group; TG1, test group 1 (1.5 g HMOs/L); TG2, test group 2 (1.5 g HMOs/L); HMG, human milk-fed group; HMOs, human milk oligosaccharides.

At baseline, no significant differences in alpha diversity indexes were observed among the formula-fed groups (all pairwise *P* > 0.05; Figure 2A and Supplementary Figures 1, 2). At 3 months of age, gene and genus Shannon index were lower in TG1 than in CG (*P* < 0.05) (Supplementary Figure 2). At 6 months of age, Faith’s PD, richness and Shannon index at gene, MGS, and genus level were lower in TG1 than in CG (all *P* < 0.05), approaching HMG (Figure 2A and Supplementary Figures 1, 2). In TG2, richness and Shannon index at the genus level were lower than those in CG at 6 months of age (*P* < 0.05), approaching HMG. All alpha diversity indexes in HMG were significantly lower than those in each of the three formula-fed groups at each timepoint (Figure 2A and Supplementary Figures 1, 2).

Beta diversity analysis, based on weighted UniFrac and PERMANOVA and visualized with PCoA, revealed a difference in gut microbiota composition among the four groups at all three timepoints (PERMANOVA, *P* ≤ 0.005, Figure 2B and Supplementary Table 1). In an analysis of the three formula-fed groups without HMG, no significant difference was observed at baseline (PERMANOVA, *P* = 0.838). However, significant differences were observed among the formula-fed groups at 3 and 6 months of age (*R*^2^ = 1.6%, *P* < 0.001 and *R*^2^ = 3.0%, *P* < 0.001, respectively). Between group comparisons showed that test groups and HMG were significantly different from CG at both post-baseline timepoints. Each test group was significantly different from HMG at baseline and 3 months, but TG1 not at 6 months (TG2 remained significantly different at 6 months, albeit the level of significance decreased with time from *P* < 0.001 to *P* = 0.04), indicating that the test groups transitioned toward HMG (Figure 2B and Supplementary Table 1).

**FIGURE 3:**
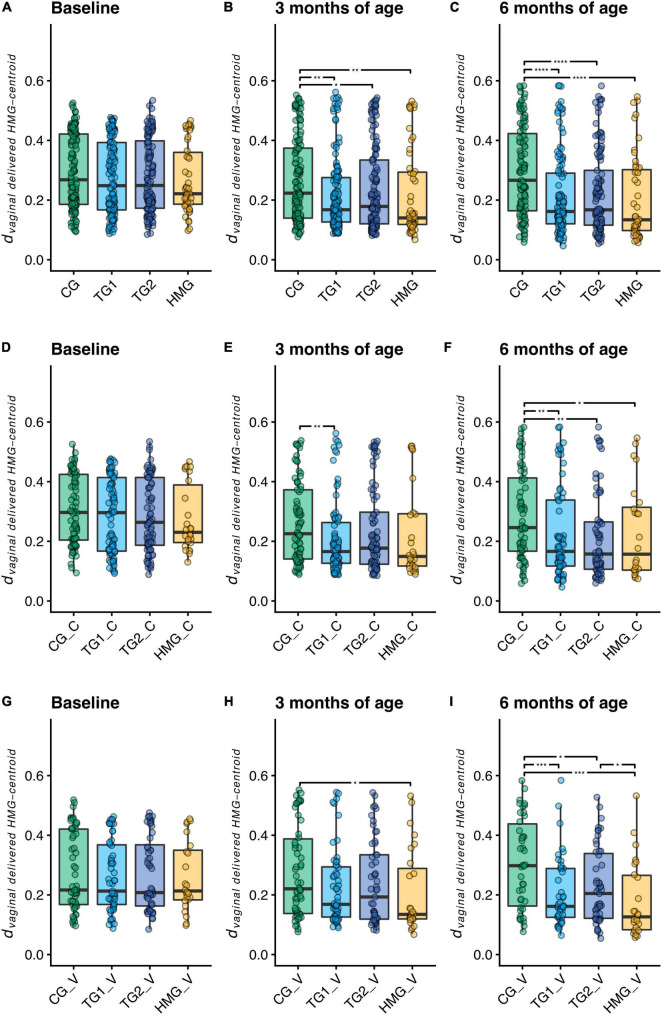
Distance to the centroid of vaginally delivered HMG infants in principal coordinates analysis space of weighted UniFrac distance (d_vaginal delivered HMG–centroid_) at each timepoint for all infants **(A–C)**, cesarean-delivered infants (**D–F**, indicated with “_C” in the labels), and vaginally delivered infants (**G–I**, indicated with “_V” in the labels). Box plots show the median and 25^th^ and 75^th^ percentiles with Tukey whiskers. Within each timepoint, all groups were compared pairwise with Mann–Whitney U tests, and significant differences are highlighted with significance bars. *P < 0.05, **P < 0.01, ***P < 0.001, ****P < 0.0001. At baseline/3/6 month of age, CG, n = 135/135/111; TG1, n = 140/138/113; TG2, n = 136/140/117; HMG, n = 50/55/50 for all infants **(A–C)**, CG, n = 79/77/69; TG1, n = 81/82/66; TG2, n = 80/85/66; HMG, n = 26/28/24 for cesarean-delivered infants **(D–F)**, CG, n = 56/58/42; TG1, n = 59/56/47; TG2, n = 56/55/51; HMG, n = 24/27/26 for vaginally delivered infants **(G–I)**. CG, control group; TG1, test group 1 (1.5 g HMOs/L); TG2, test group 2 (1.5 g HMOs/L); HMG, human milk-fed group; HMOs, human milk oligosaccharides.

To assess microbiota similarities of formula-fed infants with vaginally delivered human milk-fed infants, we calculated the phylogenetic distance between each sample and the centroid of the vaginally delivered HMG at the same timepoint (*d*_*vaginal delivered HMG*–*centroid*_; Figures 3A–C). At baseline, we did not detect differences in *d*_*vaginal delivered HMG*–*centroid*_ between the formula-fed groups (all pairwise *P* > 0.05). At 3 months, both TG1 (*P* = 0.001) and TG2 (*P* = 0.022) were closer to the vaginally delivered HMG centroid than CG, which was further strengthened at 6 months (both TG1 and TG2; *P* < 0.0001), indicating that the test groups shifted their microbiota composition toward vaginally delivered HMG. We next performed the same analysis on the subsets of cesarean- and vaginally delivered infants (Figures 3D–I). At baseline, *d*_*vaginal delivered HMG*–*centroid*_ was similar for the formula-fed groups in both subsets. At 3 months, we detected no differences between the vaginally delivered formula-fed groups and a lower *d*_*vaginal delivered HMG*–*centroid*_ in cesarean-delivered TG1 compared to cesarean-delivered CG (*P* = 0.004). At 6 months, in both delivery subsets, *d*_*vaginal delivered HMG*–*centroid*_ was significantly lower in both TG1 and TG2 than in CG (both *P* < 0.05; Figures 3F,I), indicating that the five HMO formulas shifted the gut microbiome composition of both cesarean- and vaginally delivered infants toward that of vaginally delivered human milk-fed infants.

**FIGURE 4:**
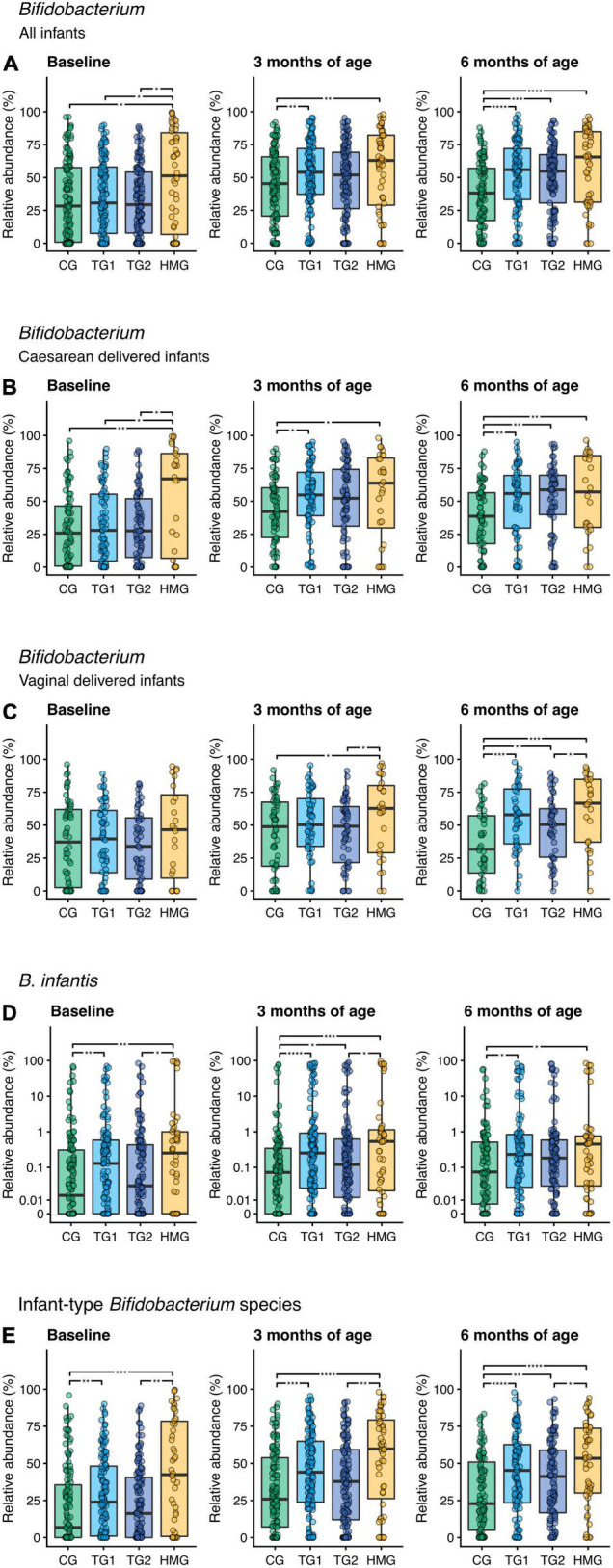
Relative abundance of **(A)** *Bifidobacterium* including all infants, **(B)** *Bifidobacterium* in cesarean-delivered infants, **(C)** *Bifidobacterium* in vaginally delivered infants, **(D)** *Bifidobacterium longum* subsp. *infantis* (*B. infantis*), and **(E)** infant-type *Bifidobacterium* species in the four feeding groups at each timepoint (baseline left, 3 months of age center, 6 months of age right). Infant-type *Bifidobacterium* species is defined as the summarized relative abundance of *B. longum* subsp. *infantis*, *B. longum* subsp. *longum*, *B. bifidum*, and *B. breve* (36). Box plots show the median and 25^th^ and 75^th^ percentiles with Tukey whiskers. Relative abundance of B. infantis is plotted on a pseudo-logarithmic scale to display values spanning several orders of magnitude, as well as zeros. Within each timepoint, all feeding groups were compared pairwise (Dunn’s test), and significant differences are highlighted with significance bars. *P < 0.05, **P < 0.01, ***P < 0.001, ****P < 0.0001. At baseline/3/6 month of age, CG, n = 135/135/111; TG1, n = 140/138/113; TG2, n = 136/140/117; HMG, n = 50/55/50 for all infants **(A,D,E)**, CG, n = 79/77/69; TG1, n = 81/82/66; TG2, n = 80/85/66; HMG, n = 26/28/24 for cesarean-delivered infants **(B)**, CG, n = 56/58/42; TG1, n = 59/56/47; TG2, n = 56/55/51; HMG, n = 24/27/26 for vaginally delivered infants **(C)**. CG, control group; TG1, test group 1 (1.5 g HMOs/L); TG2, test group 2 (1.5 g HMOs/L); HMG, human milk-fed group; HMOs, human milk oligosaccharides.

We compared abundances of predefined bacteria taxa (*Bifidobacterium* [including infant-type *Bifidobacterium* (sub)species], *Streptococcus*, *Lactobacillus*, Clostridia, and Peptostreptococcaceae; Figure 4 and Supplementary Figure 3). The relative abundance of *Bifidobacterium* seemingly increased in TG1 and TG2 over 6 months, while in CG, it decreased between 3 and 6 months. Consequently, at 6 months of age, the relative abundance of *Bifidobacterium* was higher by ∼45% in TG1 and TG2 compared to CG (*P* < 0.001) and was similar to that in HMG (*P* > 0.05; Figure 4A). Separated by delivery mode, a similar pattern was observed, particularly in the cesarean-delivered infants (Figures 4B,C). Of the *Bifidobacterium* (sub)species showing statistical differences among formula groups, *Bifidobacterium longum* subsp. *infantis* (*B. infantis*) relative abundance at 3 and 6 months of age was higher in TG1 (*P* < 0.0001 and *P* = 0.010, respectively) and TG2 (*P* = 0.016 and *P* = 0.083, respectively) than in CG and approaching HMG. However, relative abundance of *B. infantis* was also higher in TG1 than in CG at baseline (*P* = 0.006; Figure 4D). Relative abundance of infant-type *Bifidobacterium* species was higher in HMG than in CG at all timepoints (all *P* < 0.05). At 3 months, TG1 was higher than CG (*P* < 0.001), and at 6 months of age, both TG1 and TG2 were significantly higher than CG, while TG1 was indifferent compared to HMG (Figure 4E). At 6 months, abundances of *B. catenulatum* subsp. *kashiwanohense* and *B. pseudocatenulatum* were higher in TG2 than in CG and TG1 (*P* < 0.05; Supplementary Figures 4E,H). In addition, *B. dentium* abundance was significantly higher in TG2 than in CG at all three timepoints (Supplementary Figure 4F). At baseline, we did not observe any significant differences among the three formula-fed groups for Clostridia, *Lactobacillus*, Peptostreptococcaceae, and *Streptococcus* (all pairwise *P* > 0.05). At 3 and/or 6 months, the abundance of these taxa in TG1 and/or TG2 were significantly different from that of CG (*P* < 0.05) and approaching HMG (Supplementary Figure 3).

**FIGURE 5:**
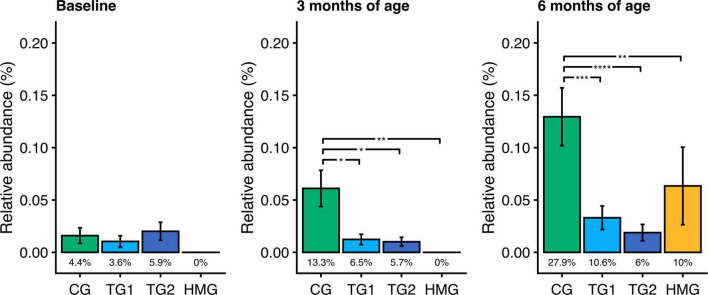
Relative abundance of toxigenic *Clostridioides difficile* in the four feeding groups at each timepoint (baseline left, 3 months of age center, 6 months of age right). Bars show the mean relative abundance with error bars indicating the standard error. Prevalence for each group (percentage of infants with detectable levels of toxigenic *C. difficile*) is displayed below the bar. Within each timepoint, the relative abundance of *C. difficile* in all feeding groups were compared pairwise (Dunn’s test), and significant differences are highlighted with significance bars. *P < 0.05, **P < 0.01, ***P < 0.001, ****P < 0.0001. At baseline/3/6 months of age, CG, *n* = 135/135/111; TG1, *n* = 140/138/113; TG2, *n* = 136/140/117; HMG, *n* = 50/55/50. CG, control group; TG1, test group 1 (1.5 g HMOs/L); TG2, test group 2 (1.5 g HMOs/L); HMG, human milk-fed group; HMOs, human milk oligosaccharides.

At baseline, the relative abundance and prevalence of toxigenic *Clostridioides (C.) difficile* were similar in the three formula-feeding groups (*P* > 0.05; Figure 5A). By contrast, at age 3 and 6 months, the relative abundances in TG1 and TG2 were lower than those in CG by 75–85% (all *P* < 0.05) and comparable to HMG (Figures 5B,C). At age 3 months, the prevalence of toxigenic *C. difficile* trended to be lower in TG1 (6.5%) and TG2 (5.7%) than in CG (13.3%; *P* ≤ 0.069). At 6 months, toxigenic *C. difficile* prevalence was lower in TG1 (10.6%) and TG2 (6.0%) than in CG (27.9%; *P* ≤ 0.001) and comparable with HMG (10.0%; *P* > 0.05). Other pathogens, such as *Campylobacter jejuni*, *Campylobacter coli*, *Clostridium perfringens*, enteropathogenic *Escherichia coli*, enterotoxigenic *Escherichia coli*, *Klebsiella pneumoniae*, and *Salmonella enterica*, had low prevalence (≤ 4 infants/feeding group) and were not investigated further.

### Fecal Biomarkers

**FIGURE 6:**
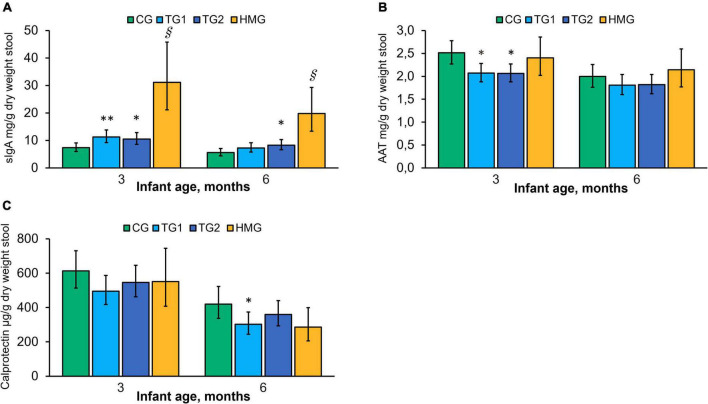
Concentration of sIgA **(A)**, AAT **(B)**, and calprotectin **(C)** in the four feeding groups at 3 and 6 months of age. Data presented as adjusted means with the 95% CI as whiskers and expressed per gram fecal dry weight. Within each timepoint, all feeding groups were compared pairwise using ANCOVA models adjusted for baseline value of the measure of interest. *P < 0.05 vs. CG; **P < 0.01 vs. CG; ^§^ *P* < 0.001 vs. all formula groups. At 3/6 months of age, CG, *n* = 102/85; TG1, *n* = 110/90; TG2, *n* = 113/99; HMG, *n* = 35/37 for sIgA; CG, *n* = 102/85; TG1, *n* = 109/88; TG2, *n* = 112/99; HMG, *n* = 34/36 for AAT; CG, *n* = 102/85; TG1, *n* = 110/89; TG2, *n* = 113/99; HMG, *n* = 35/37 for calprotectin. AAT, alpha-1-antitrypsin; CG, control group; TG1, test group 1 (1.5 g HMOs/L); TG2, test group 2 (1.5 g HMOs/L); HMG, human milk-fed group; HMOs, human milk oligosaccharides; sIgA, secretory IgA.

Adjusted mean concentrations of sIgA, AAT, and calprotectin at 3 and 6 months are shown in Figure 6. At 3 months, concentrations of sIgA in TG1 and TG2 were 53% (*P* < 0.01) and 43% (*P* < 0.05) higher than those in CG, respectively, and the difference persisted at 6 months for TG2 (*P* < 0.05). HMG had the highest sIgA concentration at both post-baseline timepoints compared to the formula-fed groups (all pairwise P < 0.001). AAT was lower in TG1 and TG2 than in CG at 3 months (*P* < 0.05). No significant difference was found at 6 months. The concentration of AAT in HMG was not statistically different from the formula-fed groups at either timepoint. Calprotectin tended to be lower in TG1 than in CG at 3 months (*P* = 0.088). At 6 months, calprotectin was lower in TG1 vs. CG (*P* < 0.05) and tended to be lower in HMG vs. CG (*P* = 0.059).

### Fecal pH and Organic Acids

Fecal pH in the test groups was lower than that in CG at 3 and 6 months (*P* < 0.05; Table 2). At 6 months, HMG showed the lowest pH (all pairwise *P* < 0.05), and pH in TG2 was lower than in TG1 (*P* < 0.05). At both post-baseline timepoints, CG had a significantly lower concentration of lactate than the other groups (Table 2). At 6 months, HMG had the highest lactate concentration (all pairwise *P* < 0.05). The relative proportion of acetate to total SCFA at 3 and 6 months was lower in CG vs. test groups (*P* < 0.01, except CG vs. TG1 at 6 months *P* = 0.059) and highest in HMG (all pairwise *P* < 0.001). At 3 months, relative proportions of butyrate and isobutyrate were lower in TG1 and TG2 than in CG (*P* < 0.05) and similar as in HMG. At 6 months, a similar pattern was observed with HMG being lowest (all pairwise *P* < 0.05), but butyrate in TG1 was no longer different from CG and isobutyrate only trended to be lower in TG1 vs. CG (*P* = 0.060). At 3 and 6 months, the proportion of propionate was not significantly different in the formula-fed groups but was significantly lower in HMG. At 3 months, the relative proportion of isovalerate was significantly higher in CG than in TG1, TG2, and HMG. At 6 months, CG trended to be higher than TG1 (*P* = 0.052) and was higher than TG2 and HMG (*P* < 0.001). At 3 months, TG1 had significantly lower valerate proportion than CG and HMG. At 6 months, CG was significantly higher than TG1 and HMG, and TG2 was significantly higher than HMG.

**TABLE 2:** Fecal pH, total concentration of lactate, and relative proportion of individual short-chain fatty acids at 3 and 6 months of age.

	3 months				6 months			
	
	CG	TG1	TG2	HMG	CG	TG1	TG2	HMG
Fecal pH^1^	6.49^a^ [6.30; 6.68]	6.05^b^ [5.87; 6.24]	5.82^b^ [5.63; 6.00]	5.95^b^ [5.66; 6.24]	6.57^a^ [6.34; 6.81]	6.14^b^ [5.90; 6.37]	5.66^c^ [5.44; 5.88]	5.19^d^ [4.85; 5.52]
Lactate (umol/g)^1^	2.38^a^ [1.69; 3.35]	4.64^b^ [3.34; 6.44]	4.73^b^ [3.41; 6.57]	4.63^b^ [2.81; 7.63]	0.68^a^ [0.44; 1.07]	1.98^b^ [1.29; 3.04]	1.77^b^ [1.18; 2.66]	4.58^c^ [2.46; 8.53]
Acetate (%)^2^	77.1 (12.0)^a^ 32.6; 98.4	81.8 (9.8)^b^ 53.7; 99.4	81.2 (10.7)^b^ 43.9; 99.1	89.2 (9.6)^c^ 54.7; 99.3	75.3 (10.9)^a^ 44.4; 94.3	77.4 (12.3)^a,b^ 8.6; 99.5	79.4 (9.8)^b^ 41.2; 97.5	86.9 (11.6)^c^ 44.2; 98.7
Butyrate (%)^3^	2.76^a^ [2.20; 3.47]	1.69^b^ [1.32; 2.17]	1.94^b^ [1.54; 2.43]	1.41^b^ [1.00; 1.98]	3.73^a^ [2.97; 4.70]	2.59^a,b^ [1.93; 3.47]	2.47^b^ [1.95; 3.13]	1.43^c^ [0.94; 2.16]
Isobutyrate (%)^3^	0.66^a^ [0.53; 0.83]	0.34^b^ [0.27; 0.42]	0.32^b^ [0.26; 0.40]	0.25^b^ [0.19; 0.34]	0.80^a^ [0.64; 1.01]	0.60^a^ [0.47; 0.76]	0.37^b^ [0.29; 0.47]	0.25^c^ [0.17; 0.36]
Propionate (%)^3^	12.6^a^ [11.0; 14.5]	10.4^a^ [9.0; 12.0]	10.4^a^ [8.9; 12.2]	3.7^b^ [2.6; 5.3]	13.9^a^ [12.1; 16.1]	12.1^a^ [10.0; 14.6]	12.6^a^ [11.1; 14.3]	5.7^b^ [4.2; 7.7]
Isovalerate (%)^3^	0.53^a^ [0.43; 0.66]	0.28^b^ [0.23; 0.35]	0.28^b^ [0.23; 0.33]	0.27^b^ [0.21; 0.36]	0.58^a^ [0.46; 0.73]	0.43^a^ [0.34; 0.55]	0.29^b^ [0.23; 0.36]	0.22^b^ [0.17; 0.29]
Valerate (%)^2^	0.33 (0.91)^a^ 0.02; 6.65	0.21 (0.48)^b^ 0.02; 3.91	0.23 (0.79)^a,b^ 0.03; 8.11	0.31 (0.41)^a^ 0.05; 2.64	0.37 (0.72)^a^ 0.04; 5.13	0.27 (0.70)^b,c^ 0.02; 5.96	0.30 (0.60)^a,b^ 0.03; 3.85	0.23 (0.37)^c^ 0.04; 1.76

*^1^Values are adjusted means [95% confidence intervals]. For lactate, absolute concentrations in the feces based on dry matter are reported.*

*^2^Values shown are mean (SD); Min, Max for the relative proportion to total short-chain fatty acid concentration.*

*^3^Values shown are geometric means [95% confidence intervals] for the relative proportion to total short-chain fatty acid concentration.*

*Values without a common superscript letter within one timepoint are significantly different from each other (P < 0.05 based on pairwise comparison using ANCOVA models adjusted for baseline value of the measure of interest or Wilcoxon test for valerate). At 3/6 month of age, CG, n = 127/101; TG1, n = 133/102; TG2, n = 135/113; HMG, n = 56/51 for fecal pH; CG, n = 115/89; TG1, n = 125/95; TG2, n = 125/105; HMG, n = 54/45 for lactate; CG, n = 114/81; TG1, n = 122/84; TG2, n = 119/98; HMG, n = 54/46 for acetate, butyrate, isobutyrate, propionate, isovalerate, and valerate. CG, control group; TG1, test group 1 (1.5 g HMOs/L); TG2, test group 2 (1.5 g HMOs/L); HMG, human milk-fed group; HMOs, human milk oligosaccharides.*

## Discussion

To our knowledge, this is the first study investigating the effect of an infant formula with a specific blend of five HMOs on gut maturation, including microbiota composition and metabolism, as well as gut barrier and immune function. Our main findings are the ability of this specific HMO blend to drive the gut microbiota development of formula-fed infants directionally toward that of breastfed infants, including higher bifidobacteria and lower toxigenic *C. difficile* abundance, and to improve early-life intestinal immune response as indicated by the higher fecal sIgA concentration in the test groups vs. CG. Our results unveil important roles of the tested HMO blend in the development of the gut microbiome in early life. Given the importance the gut microbiome has in regulating and fine-tuning the development of the immune system, the tested HMO blend can potentially contribute beneficially to the short- and long-term health of formula-fed infants.

The HMO formulas impacted the gut microbiota composition as early as 3 months of age as indicated by beta diversity analysis. At 6-month age, gut microbiota modulating effects were even more distinct with some of the alpha-diversity indexes (richness and Shannon index at the genus level) being significantly lower in the test groups than in CG, with TG1 being no longer significantly different from HMG based on beta diversity analysis. Our data also show that the shift of the test groups toward the vaginally delivered HMG was observed irrespectively of the delivery mode, which is an interesting observation considering the reference role the vaginally delivered breastfed babies play in the interpretation of the gut microbiota findings.

The findings at 6 months suggest that HMOs play a crucial role in shaping the gut microbiome even after introduction of complementary foods and that the five-HMO blend promotes a human milk-fed alike bifidobacteria pattern beyond the exclusive formula-feeding period. *Bifidobacterium* species found in the gastrointestinal tract of infants are known to metabolize HMOs (37). A noteworthy difference in our study was the higher abundances of *B. infantis* in the test groups than in CG. These results indicate that *B. infantis* gains a competitive advantage in the presence of the five HMOs. *B. infantis* is expected to have the genetic makeup to use the dominant oligosaccharides (38), and several strains were shown to broadly metabolize HMOs like those used in this study (39, 40). Interestingly, the effects of the HMO-supplemented formula on the gut microbiota in cesarean- or vaginally born infants were similar, changing the microbiota toward the composition observed in vaginally born infants of HMG, including an increase in *Bifidobacterium*. This suggests that HMOs help correct some of the potential underlying dysbiosis in cesarean-born delivered infants (41). A similar beneficial effect on dysbiotic gut microbiota in cesarean-born delivered infants has been reported for breastfeeding (41, 42). A previous study also found a bifidogenic effect in infants receiving formula with two HMOs (2′FL, LNnT) which was more pronounced in the cesarean-born infants. However, the study did not find any specific effect on *B. infantis* (24).

In our study, acetate proportion and lactate concentration were higher in the infants receiving the HMO formulas than those of CG. Since acetate and lactate are the main end-products of the bifidobacteria catabolism (43), our results can be explained by the increased growth of bifidobacteria in the test groups and possibly also by HMO-stimulated increased activity of enzymes involved in the metabolic pathways of bifidobacteria (44). On the other hand, we observed that proportions of butyrate, isobutyrate, or isovalerate were lower in the test groups and HMG than in CG, indicating a more diverse microbiota in CG, for example, butyrate is produced by *Bacteroides* and Firmicutes (e.g., *Clostridium*), but not by *Bifidobacterium* (45). Lactate and acetate play a vital role for the pH regulation in the colon (46), and the lower pH observed in the test groups in our study possibly contributed to the reduction in *C. difficile* in the test groups. Acetate might also have contributed directly to the lower relative abundance of *C. difficile* as results from an *in vivo* study indicate that acetate promotes innate host responses against *C. difficile* through coordinated action on neutrophils and group 3 innate lymphoid cells (47). A direct effect of the HMOs preventing epithelial adhesion of *C. difficile* by acting as decoy receptors might have been possible too; however, pre-clinical work suggests that the effect is rather indirect *via* gut microbiota modulation (48). Our findings about toxigenic C. *difficile* are of importance because they indicate that the five-HMO blend can reduce a risk factor for infectious diarrhea in formula-fed infants.

The intestinal immune response, gut barrier function/permeability, and the inflammatory signals in the gut are evolving during gut maturation. Fecal sIgA, a marker of intestinal immune response (49), was higher in TG1 and TG2 than in CG at 3 months, and the difference persisted in TG2 at 6 months. This may be linked to the increases in bifidobacteria, which have been shown to interact with human immune cells (50), and their surface-associated molecules and their metabolites may exert immunomodulatory functions (51, 52). Recently, Laursen and colleagues demonstrated that human milk-promoted *Bifidobacterium* species produce indole-3-lactate, a tryptophan metabolite, which may impact intestinal homeostasis and immune response in early life (36). In our study, test groups had significantly higher abundance of these breastmilk-promoted *Bifidobacterium* species, indicating that the five-HMO blend likely increases the production of some immunomodulatory metabolites in formula-fed infants. Furthermore, acetate has been shown to support T-cell-dependent intestinal IgA production in mice (53), and bifidobacteria-supplemented infant formula has been associated with increased fecal sIgA (54, 55). In addition to the sIgA produced by the B lymphocytes in the submucosal tissues, sIgA is also provided to infants *via* consumption of human milk (56), which is consistent with our finding that the HMG had a significantly higher sIgA concentration than the formula-fed groups. We observed that concentrations of AAT, a marker of intestinal permeability, were significantly lower in TG1 and TG2 than in CG at 3 months. In formula- and human milk-fed infants, AAT is known to decrease during infancy (57), and our results indicate that HMOs may contribute to this development in formula-fed infants in the first months after birth. HMG also showed the expected downward trend from 3 to 6 months, but AAT concentration was numerically higher at 6 months than that in the formula groups. This is likely due to AAT found in human milk which contributes to the overall measured concentrations but is not produced by the infant (58). We also found some indication that the HMO formulas contribute to the known decrease in the gut inflammation marker calprotectin in infants over time (59, 60). Together, our fecal biomarker results indicate that HMO-supplemented formula may be supportive of the infant intestinal immune development and gut barrier function.

Our study has several strengths. We had a substantial sample size with > 100 infants in the formula groups and > 50 in HMG. We used shotgun metagenomics which, compared with traditional 16S rRNA technique, provides more accurate and reliable profiling of bacterial (sub)species, allowing us to assess different *Bifidobacterium* (sub)species and to evaluate pathogenicity of species based on the presence of corresponding virulence genes (e.g., toxigenic *C. difficile*). To strengthen our assessment of the impact of the HMOs on infant gut physiology, we complemented our analysis with selected fecal metabolites and biomarkers. A limitation of our study is the lack of detailed data of complementary feeding after 4 months of age, which likely contributed to the gut maturation between 4 to 6 months of age. The available data show that most of the formula-fed infants have received complementary foods between 4 and 6 months of age (>90%), and there are unlikely any substantial differences in the types of consumed complementary foods among the formula-fed groups due to the randomization. Our study tested two different dosages of the five-HMO blend, but we did not observe any substantial dose-dependent response. Either the difference of 1 g HMOs per liter was not enough to detect potential dose-dependent responses or above 1.5 g HMOs per liter, there is a kind of a saturation effect for changes in microbiota and gut development using our HMO blend. The actual difference in HMOs actively used by the microbiota between the test groups might have been even smaller than 1 g per liter as the HMOs also act as decoy receptors and interact directly with the intestinal epithelium, and a small proportion is absorbed intact (7). The higher dose (TG2) doing slightly less good than TG1 for certain microbiota outcomes when compared to CG might therefore be due to data variability. Also, the proportion of HMOs not used by the microbiota might be increased with higher concentrations, which would be consistent with the findings for sIgA, where the direct interaction of HMOs with the intestinal mucosa might be more important than the prebiotic effect and for which TG2 was numerically higher than TG1 at 6-month age and significantly different from CG. Further research is needed on potential dose-dependent effects of HMOs and HMO blends.

## Conclusion

This multi-country, double-blinded trial demonstrates that the intestinal maturation of formula-fed infants can be beneficially modulated by an infant formula containing a specific blend of five HMOs. Consumption of HMO-supplemented formula in the first 6 months of life shifts the microbiota composition closer to that of infants receiving human milk. This includes a strong bifidogenic effect and less toxigenic *C. difficile*, which is expected to decrease the risk of diarrheal illness. The shift in the gut microbiota may mediate, to a certain extent, the effects that have been seen on intestinal immune response evidenced by the substantial increase in fecal sIgA. Supplementing infant formula with our blend of five HMOs is therefore a promising and efficacious approach to support the gut microbiome and gut barrier and immune maturation during early infancy of formula-fed infants.

## Data Availability Statement

The raw data supporting the conclusions of this article will be made available by the authors upon reasonable request, without undue reservation.

## Ethics Statement

This study involving human participants was reviewed and approved by the Medical Research Council (Budapest, Hungary), the Bioethics Committee at the Regional Medical Chamber (Gdańsk, Poland) and the Ethics Committee of the Scientific Research at Medical University (Sofia, Bulgaria). Written informed consent to participate in this study was provided by the participants or their legal guardian/next of kin prior to enrollment.

## 5 HMO Study Investigator Consortium

Members of the 5 HMO Study Investigator Consortium were Viktor Bauer, Dr. Kenessey Albert Hospital and Clinic, Balassagyarmat, Hungary; Malgorzata Arciszewska, Polyclinic of Gynecology and Obstetrics Arciszewscy, Bialystok, Poland; Maria Tarneva and Irina Popova, University Multiprofile Hospital for Active Treatment Deva Mariya—Neonatology, Burgas, Bulgaria; Svilen Dosev, Medical Center Prolet—Pediatrics department, Ruse, Bulgaria; Sirma Dimitrova, Medical Center Excelsior, Sofia, Bulgaria; Olga Nikolova, Multiprofile Hospital for Active Treatment Sveti Ivan Rilski, Kozloduy, Bulgaria; Marzena Nowak, Medical Center PROMED, Krakow, Poland; Magdalena Szuflinska-Sidorowicz, Medical Center Pratia Warszawa, Warszawa, Poland; Bartosz Korczowski, College of Medical Sciences, University of Rzeszów, Rzeszów, Poland; Rositsa Karcheva-Beloeva, Medical Center-1, Sevlievo, Bulgaria; Stefan Banov, Individual Practice for Specialized Medical Assistance, Stara Zagora, Bulgaria; Boguslawa Cimoszko, Primary Health Care Clinic Clinical Vitae, Gdansk, Poland; Wieslaw Olechowski, ALERGO-MED Specialist Medical Clinic, Tarnow, Poland; Robert Simko, Futurenest Clinical Research, Miskolc, Hungary; Zsuzsanna Tengelyi, Medical Center Clinexpert, Budapest, Hungary; Piotr Korbal, Dr. Jan Biziel’s University Hospital No. 2, Bydgoszcz, Poland; Marta Zolnowska, Plejady Medical Center, Krakow, Poland; Anton Bilev, Medical Center Sveti Ivan Rilski Chudotvorets, Blagoevgrad, Bulgaria; Georgios Vasilopoulos, Center of Innovative Therapies, Piaseczno, Poland; Sylwia Korzynska, Medical Center Pratia Ostroleka, Ostroleka, Poland; István Laki, Kanizsai Dorottya Hospital, Nagykanizsa, Hungary; Margarita Koleva-Syarova, Diagnostic Consultative Center Ritam, Stara Zagora, Bulgaria; Toni Grigorov, Multiprofile Hospital for Active Treatment Sveti Georgi, Montana, Bulgaria; Steliyana Kraeva, Alitera Medical Centre, Sofia, Bulgaria; Éva Kovács, Family Pediatric Surgery/Babadoki Ltd., Szeged, Hungary; Rada Markova, Policlinic Bulgaria—Department of pediatrics; Sofia, Bulgaria; Grazyna Jasieniak-Pinis, Non-public Health Care Institution Specialist Clinics ATOPIA, Krakow, Poland; Katalin Fister Bugát Pál Hospital—Department of Pediatrics, Gyöngyös, Hungary; Tatyana Stoeva, Medical Center—Izgrev Ltd., Sofia, Bulgaria.

## Author Contributions

NS, BB, and CC designed the study. MB, IT, AK, and the 5 HMO Study Investigator Consortium conducted the experiments. HP, OL, AE, DG, NS, BB, and CC analyzed the data. HP, AE, NS, BB, and CC wrote the first draft. CC had primary responsibility for the final content. All authors read and approved the final version of the manuscript.

## Conflict of Interest

This study received funding from Nestlé Nutrition, Société des Produits Nestlé S.A., Switzerland. DG, NS, BB, and CC were current employees of the funder. The funder had the following involvement with the study: study design, data analysis, decision to publish, and preparation of the manuscript. HP, OL, and AE were employees of Clinical Microbiomics, Denmark, which was involved in the sample and data analysis. The remaining authors declare that the research was conducted in the absence of any commercial or financial relationships that could be construed as a potential conflict of interest.

## Publisher’s Note

All claims expressed in this article are solely those of the authors and do not necessarily represent those of their affiliated organizations, or those of the publisher, the editors and the reviewers. Any product that may be evaluated in this article, or claim that may be made by its manufacturer, is not guaranteed or endorsed by the publisher.

## Abbreviations

AAT: alpha-1-antitrypsin
2′FL: 2′fucosyllactose
3′SL: 3′sialyllactose
6′SL: 6′sialyllactose
*B*.: *Bifidobacterium*
*C. difficile*: *Clostridioides difficile*
CG: control group
DFL: 2′,3-di-O-fucosyllactose
HMG: human milk group
HMOs: human milk oligosaccharides
IF: infant formula
LNT: lacto-N-tetraose
LNnT: lacto-N-neotetraose
MGS: metagenomic species
PCoA: principal coordinates analysis
PD: phylogenetic diversity
PERMANOVA: permutational multivariate analysis of variance
SCFA: short-chain fatty acid
sIgA: secretory immunoglobulin A
TG1: test group 1
TG2: test group 2.
