# Supplementary material for: Infant Formula With a Specific Blend of Five Human Milk Oligosaccharides Drives the Gut Microbiota Development and Improves Gut Maturation Markers: A Randomized Controlled Trial
Source: Front Nutr. 2022 Jul 6;9:920362. doi: 10.3389/fnut.2022.920362 (PMC9298649; doi:10.3389/fnut.2022.920362)
Supplement: Supplementary file 1 [file Data_Sheet_1.DOCX]

**Online supplementary material**

**Supplementary Methods**

Table of Contents

[DNA extraction and sequencing 1](#_Toc86667176)

[Data preprocessing 1](#_Toc86667177)

[Gene catalog and MGS definitions 2](#_Toc86667178)

[Mapping reads to gene catalog 2](#_Toc86667179)

[Taxonomical annotation of MGSs 2](#_Toc86667180)

[MGS relative abundance calculation 3](#_Toc86667181)

[Derivation of MGS-based species tree 3](#_Toc86667182)

[Detection of pathogenic species 4](#_Toc86667183)

# DNA extraction and sequencing

Microbial DNA was extracted and purified from frozen faeces using the NucleoSpin Soil kit (Macherey-Nagel, Dueren, Germany). Lysis was performed by bead beating horizontally on a Vortex-Genie 2 at 2700 rpm for 5 minutes. Library preparation and next-generation sequencing were performed at Novogene (Cambridge, UK). The purified genomic DNA was randomly sheared into fragments of ~350 base pairs (bp) and used for library construction using the NEBNext Ultra Library Prep Kit for Illumina (New England Biolabs, Ipswich, USA). Libraries were evaluated using Qubit 2.0 fluorometer (Thermo Fisher Scientific, Waltham, USA) quantitation and Agilent 2100 Bioanalyzer (Agilent, Santa Clara, USA) for the fragment size distribution. Quantitative real-time PCR was used to determine the concentration of the final library prior to sequencing. Paired-end 2×150 bp sequencing was performed on an Illumina NovaSeq 6000 (Illumina, San Diego, USA).

# Data preprocessing

Raw FASTQ files were filtered to remove human genomic contamination by discarding read pairs in which either read mapped to the human reference genome GRCh38 with Bowtie2 (v. 0.2.3.4.1) [1]. Reads were then trimmed to remove adapters and bases with a Phred score below 20 using AdapterRemoval (v. 2.2.4) [2].

# Gene catalog and MGS definitions

The Clinical Microbiomics in-house infant fecal microbiome gene catalog (containing 23,968,023 microbial genes) was used for reference gene catalog and the corresponding set of 1306 metagenomics species (MGS) definitions for MGS abundance profiling. The MGSs were built based on >5000 deep-sequenced human adult and infant gut samples using an approach based on the metagenomic species concept [3] and have highly coherent abundance and base composition in a set of 1776 independent reference human gut samples.

# Mapping reads to gene catalog

Trimmed, human-filtered reads were mapped to the gene catalog using BWA mem (v. 0.7.16a) [4]. An individual read was considered mapped to a gene if the mapping quality (MAPQ) was ≥20 and the read aligned with ≥95% identity over ≥100 bp. However, if >10 bases of the read did not align to the gene or extend beyond the gene, the read was considered unmapped. Reads meeting these criteria except for the MAPQ threshold were considered multi-mapped. Each read pair was counted as either 1) mapped to a specific gene, if one or both individual reads mapped to a gene, or 2) multi-mapped, if neither read was mapped, and at least one was multi-mapped, or 3) unmapped, if neither individual read mapped. If the two reads each mapped to a different gene, the gene mapped by read 1 was counted but not the gene mapped by read 2. The resulting gene count table, of number of mapped read pairs for each gene, was used to calculate the relative abundance of each MGS.

# Taxonomical annotation of MGSs

MGSs were annotated by blasting catalog genes to the NCBI RefSeq genome database (2020-01-27) using a minimum of 80% sequence coverage with varying levels of similarity: 95, 95, 85, 75, 65, 55, 50, and 45% for gene taxonomy annotation at subspecies, species, genus, family, order, class, phylum, and superkingdom level, respectively. To assign species, genus, family, order, class, and phylum or superkingdom level taxonomy to an MGS, we required 75, 60, 50, 40, 30, and 25%, respectively, of its genes to be consistently annotated to the same taxa at the given level. Furthermore, for species and at genus level annotation, we required that less than 10% of the remaining MGS genes to be annotated to any alternative taxon. Finally, we applied CheckM to each MGS [5], and updated our annotations with CheckM annotation for 118 MGSs for which CheckM provided annotation at higher resolution (lower taxonomic rank).

# MGS relative abundance calculation

For each MGS, a signature gene set had been previously defined as the 100 genes optimized for accurate abundance profiling of the MGS. An MGS count table was created by counting the number of reads mapped to the MGS signature genes per sample. An MGS was considered detected if reads from a sample mapped to at least three of its signature genes; measurements that did not satisfy this criterion were set to zero. Based on internal benchmarks, this threshold results in 99.6% specificity. The MGS count table was normalized according to effective gene length and then normalized sample-wise to sum to 100%, resulting in relative abundance estimates for each MGS.

All alpha and beta diversity calculations were based on downsampled (rarefied) MGS abundance profiles to control for uneven sampling. These were calculated by random sampling, without replacement, of a fixed number of signature gene counts per sample, and then following the procedure described above.

# Derivation of MGS-based species tree

The species tree for the MGS was created based on single-copy bacterial and archaeal marker genes from the Genome Taxonomy Database (GTDB) consisting of 120 bacterial and 122 archaeal marker-genes belonging to either TIGRFAM or PFAM protein families [6, 7]. First, INTERPROSCAN [8] was used to identify marker genes within each MGS. Multi-copy marker genes and marker genes that were identified in < 10 MGS were excluded, resulting in a total of 111 bacterial and 26 archaeal marker genes with sufficient coverage. 7 of the 130 marker genes were shared between bacteria and archaea. MGSs with fewer than 10 marker genes identified by this method and MGS that were annotated as eukaryotes were excluded. Protein sequences from these 130 marker genes were aligned using HMMalign (v.3.2.1), and non-aligned residues were trimmed from the multiple sequence alignment. The species tree was next inferred using the concatenation-based species tree approach in IQtree [9] with 1000 ultrafast bootstraps [10] and an edge-linked partition model [11]. The species tree covered a total of 1,255 MGS and was rooted with archaea as an outgroup.

# Detection of pathogenic species

Presence of pathogenic strains of *Campylobacter jejuni*, *Campylobacter coli*, *Clostridioides (Clostridium) difficile*, *Clostridium perfringens*, *Escherichia coli* (EPEC and ETEC), *Klebsiella pneumoniae*, and *Salmonella enterica* was inferred based on the presence of specific virulence genes (**Listing 1**). For all but *S. enterica*, we located these virulence genes in the gene catalog using BLASTN, requiring 90% identity over an alignment covering of at least 75% of the reference gene. A virulence gene was considered detected in a sample if the mapped reads covered at least 85% of the gene. A pathogenic strain was considered detected if all the listed virulence genes for that species were detected.

However, we used a different approach for *S. enterica* because its genes (particularly the virulence genes) were not detected in the gene catalog. Here, raw reads were mapped directly to the reference genes using "kma" [12]. Kma-mapped hits with 90% identity and an alignment length of at least 75% of the template gene length were selected. *S. enterica* was considered detected if the majority of the genes present on the pathogenicity islands SPI-1 and SPI-2 were detected.

Listing 1: Virulence factors for the pathogenic species of interest. Accession number lists a reference genome accession number and the position in the genome.

| Pathogen | Virulence factor | Accession number | Reference |
| --- | --- | --- | --- |
| *Campylobacter* | Cytolethal-distending-toxin (cdtA) | NC_002163.1 (90264 - 91070) | [13] |
|  | Cytolethal-distending-toxin (cdtB) | NC_002163.1 (89470 - 90267) |  |
|  | Cytolethal-distending-toxin (cdtC) | NC_002163.1 (88890 - 89459) |  |
| *Clostridioides difficile* | Exotocin (toxA) | NC_009089.1 (795843 - 803975) | [14] |
|  | Exotoxin (toxB) | NC_009089.1 (787393 – 794493) |  |
| *Clostridium perfringens* | Enterotoxin (cpe) | CP000312.1 (464981 - 465940) | [15] |
| *Escherichia coli EPEC* | Intimin receptor (tir) | FM180568.1 (4113830 - 4115482) | [16] |
|  | Intimin (eae) | FM180568.1 (4110343 - 4113162) |  |
| *Escherichia coli ETEC* | Heat-stable toxin (ST) | NC_013507.1 (28176 - 28394) | [13] |
|  | Heat-labile toxin (LT) | NC_017722.1 (50594 – 51370, 50223 – 50597) |  |
| *Klebsiella pneumoniae* | Regulator of the mucoid phenotype (rmpA) | NC_012731.1 (3444784 - 3446416) | [17] |
|  | Mucoviscosity-associated gene A (magA) | NC_012731.1 (3534669 - 3535895) | [18] |
| *Salmonella enterica* | SPI-1 (invasion) | NC_003198.1 (2858736 - 2900586) |  |
|  | SPI-2 (intracellular survival) | NC_003198.1 (1624920 - 1666524) | [19] |

References:

1. Langmead, B. and S.L. Salzberg, *Fast gapped-read alignment with Bowtie 2.* Nat Methods, 2012. **9**(4): p. 357-9.

2. Schubert, M., S. Lindgreen, and L. Orlando, *AdapterRemoval v2: rapid adapter trimming, identification, and read merging.* BMC Res Notes, 2016. **9**: p. 88.

3. Nielsen, H.B., et al., *Identification and assembly of genomes and genetic elements in complex metagenomic samples without using reference genomes.* Nat Biotechnol, 2014. **32**(8): p. 822-8.

4. Li, H. and R. Durbin, *Fast and accurate short read alignment with Burrows-Wheeler transform.* Bioinformatics, 2009. **25**(14): p. 1754-60.

5. Parks, D.H., et al., *CheckM: assessing the quality of microbial genomes recovered from isolates, single cells, and metagenomes.* Genome Res, 2015. **25**(7): p. 1043-55.

6. Parks, D.H., et al., *A complete domain-to-species taxonomy for Bacteria and Archaea.* Nat Biotechnol, 2020. **38**(9): p. 1079-1086.

7. Parks, D.H., et al., *A standardized bacterial taxonomy based on genome phylogeny substantially revises the tree of life.* Nat Biotechnol, 2018. **36**(10): p. 996-1004.

8. Jones, P., et al., *InterProScan 5: genome-scale protein function classification.* Bioinformatics, 2014. **30**(9): p. 1236-40.

9. Nguyen, L.T., et al., *IQ-TREE: a fast and effective stochastic algorithm for estimating maximum-likelihood phylogenies.* Mol Biol Evol, 2015. **32**(1): p. 268-74.

10. Hoang, D.T., et al., *UFBoot2: Improving the Ultrafast Bootstrap Approximation.* Mol Biol Evol, 2018. **35**(2): p. 518-522.

11. Chernomor, O., A. von Haeseler, and B.Q. Minh, *Terrace Aware Data Structure for Phylogenomic Inference from Supermatrices.* Syst Biol, 2016. **65**(6): p. 997-1008.

12. Clausen, P., F.M. Aarestrup, and O. Lund, *Rapid and precise alignment of raw reads against redundant databases with KMA.* BMC Bioinformatics, 2018. **19**(1): p. 307.

13. Barer, M., et al., *Medical microbiology: A guide to microbial infections: pathogenesis, immunity, laboratory investigation and control - Nineteenth edition.* 2019, Amsterdam: Elsevier.

14. Awad, M.M., et al., *Clostridium difficile virulence factors: Insights into an anaerobic spore-forming pathogen.* Gut Microbes, 2014. **5**(5): p. 579-93.

15. Freedman, J.C., A. Shrestha, and B.A. McClane, *Clostridium perfringens Enterotoxin: Action, Genetics, and Translational Applications.* Toxins (Basel), 2016. **8**(3).

16. Kenny, B. and J. Warawa, *Enteropathogenic Escherichia coli (EPEC) Tir receptor molecule does not undergo full modification when introduced into host cells by EPEC-independent mechanisms.* Infect Immun, 2001. **69**(3): p. 1444-53.

17. Yeh, K.M., et al., *Capsular serotype K1 or K2, rather than magA and rmpA, is a major virulence determinant for Klebsiella pneumoniae liver abscess in Singapore and Taiwan.* J Clin Microbiol, 2007. **45**(2): p. 466-71.

18. Khaertynov, K.S., et al., *Virulence Factors and Antibiotic Resistance of Klebsiella pneumoniae Strains Isolated From Neonates With Sepsis.* Front Med (Lausanne), 2018. **5**: p. 225.

19. Kaur, J. and S.K. Jain, *Role of antigens and virulence factors of Salmonella enterica serovar Typhi in its pathogenesis.* Microbiol Res, 2012. **167**(4): p. 199-210.
