# Supplementary material for: Infant Formula With a Specific Blend of Five Human Milk Oligosaccharides Drives the Gut Microbiota Development and Improves Gut Maturation Markers: A Randomized Controlled Trial
Source: Front Nutr. 2022 Jul 6;9:920362. doi: 10.3389/fnut.2022.920362 (PMC9298649; doi:10.3389/fnut.2022.920362)
Supplement: Supplementary file 2 [file Table_1.DOCX]

**Online supplementary material**

#### Supplementary Tables

##### Supplementary table 1:

Significance and explained variation of permutational multivariate analysis of variance (PERMANOVA) on the indicated subset of samples.

| **Timepoint** | **Included groups** | **n** | **R^2^** | **P** |
| --- | --- | --- | --- | --- |
| Baseline | All | 461 | 1.9629 % | 0.0049950 |
| 3 month of age | All | 468 | 2.5442 % | 0.0019980 |
| 6 month of age | All | 391 | 3.8153 % | 0.0009990 |
| Baseline | Formula-fed | 411 | 0.2618 % | 0.8381618 |
| 3 month of age | Formula-fed | 413 | 1.6385 % | 0.0009990 |
| 6 month of age | Formula-fed | 341 | 2.9936 % | 0.0009990 |
| Baseline | CG vs TG1 | 275 | 0.2357 % | 0.6233766 |
| 3 month of age | CG vs TG1 | 273 | 2.0446 % | 0.0029970 |
| 6 month of age | CG vs TG1 | 224 | 3.3616 % | 0.0009990 |
| Baseline | CG vs TG2 | 271 | 0.1150 % | 0.8961039 |
| 3 month of age | CG vs TG2 | 275 | 1.2427 % | 0.0239760 |
| 6 month of age | CG vs TG2 | 228 | 2.7825 % | 0.0019980 |
| Baseline | TG1 vs TG2 | 276 | 0.2387 % | 0.5744256 |
| 3 month of age | TG1 vs TG2 | 278 | 0.3965 % | 0.3156843 |
| 6 month of age | TG1 vs TG2 | 230 | 0.4273 % | 0.3976024 |
| Baseline | HMG vs CG | 185 | 3.3035 % | 0.0019980 |
| 3 month of age | HMG vs CG | 190 | 4.1446 % | 0.0009990 |
| 6 month of age | HMG vs CG | 161 | 5.5738 % | 0.0009990 |
| Baseline | HMG vs TG1 | 190 | 3.1219 % | 0.0019980 |
| 3 month of age | HMG vs TG1 | 193 | 1.4131 % | 0.0359640 |
| 6 month of age | HMG vs TG1 | 163 | 1.3326 % | 0.0639361 |
| Baseline | HMG vs TG2 | 186 | 3.8865 % | 0.0009990 |
| 3 month of age | HMG vs TG2 | 195 | 1.7663 % | 0.0239760 |
| 6 month of age | HMG vs TG2 | 167 | 1.5336 % | 0.0389610 |

P-values are for PERMANOVA using feeding group as explanatory variable. n: lists the number of infant samples for the given timepoint. CG, Control Group; TG1, Test Group 1 (1.5 g HMOs/L); TG2, Test Group 2 (1.5 g HMOs/L); HMG, Human milk-fed group.
