# Supplementary material for: Infant Formula With a Specific Blend of Five Human Milk Oligosaccharides Drives the Gut Microbiota Development and Improves Gut Maturation Markers: A Randomized Controlled Trial
Source: Front Nutr. 2022 Jul 6;9:920362. doi: 10.3389/fnut.2022.920362 (PMC9298649; doi:10.3389/fnut.2022.920362)
Supplement: Supplementary file 5 [file Image_3.pdf]

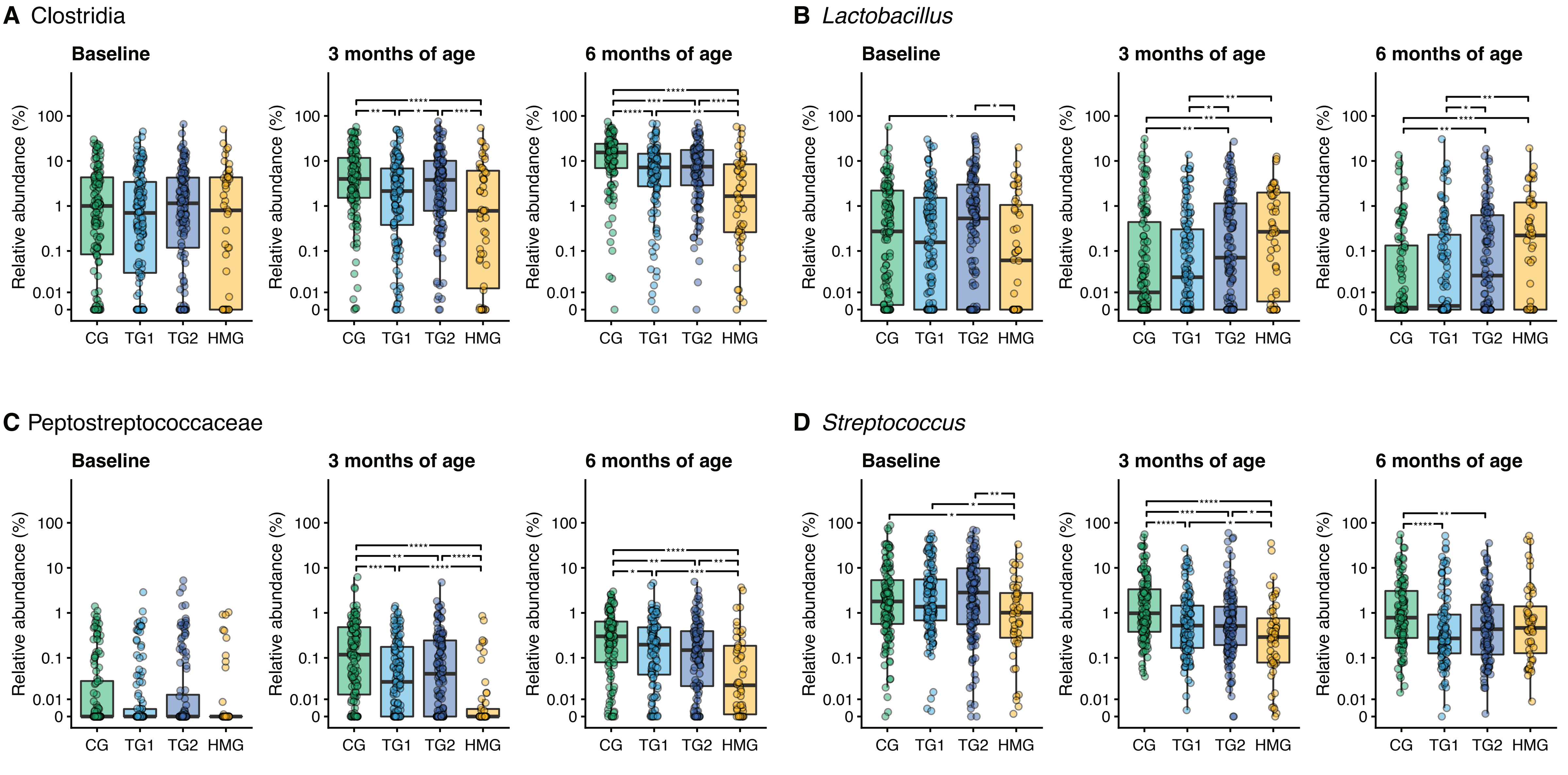

Supplementary Figure 3:
Relative abundance of selected taxa in the four feeding groups at each timepoint (baseline left, 3 months of age center, 6 months of age right). Within each timepoint, all feeding groups were compared pairwise and significant differences (Dunn's test) are highlighted with significance bars. Figures are plotted on a pseudo-logarithmic scale in order to display values spanning several orders of magnitude as well as zeros. Box plots show the median and 25th and 75th percentiles with Tukey whiskers. At baseline/3/6 month of age, CG, n=135/135/111; TG1, n=140/138/113; TG2, n=136/140/117; HMG, n=50/55/50. *: P<0.05, **: P<0.01, ***: P<0.001, ****: P<0.0001.
